# Editorial: Therapeutic and health-promoting properties of polysaccharides in personalized foods

**DOI:** 10.3389/fnut.2023.1245949

**Published:** 2023-07-12

**Authors:** Yuthana Phimolsiripol, Warintorn Ruksiriwanich, Phisit Seesuriyachan, Francisco J. Barba

**Affiliations:** ^1^Faculty of Agro-Industry, Chiang Mai University, Chiang Mai, Thailand; ^2^Center of Excellence in Agro Bio-Circular-Green Industry, Chiang Mai University, Chiang Mai, Thailand; ^3^Faculty of Pharmacy, Chiang Mai University, Chiang Mai, Thailand; ^4^Department of Preventive Medicine and Public Health, Food Science, Toxicology and Forensic Medicine, Faculty of Pharmacy, Universitat de València, Valencia, Spain

**Keywords:** therapeutic, health food, polysaccharide, personalized foods, antioxidant, cancer, microbiome

Nowadays, numerous bioactive compounds, especially polysaccharides with functional properties have been discovered, mainly those isolated from natural sources. Bioactive properties have been reported with the effects of antioxidant, antitumor, immunomodulatory, immunostimulatory, inflammatory, antinociception, anticoagulant, antiviral, antiprotozoal, antibacterial, and antihyperlipidemic. Appropriate extraction and precipitation methods, purification, and characterization of bioactive compounds have also been reported. The molecular chemical structures, functional groups, and mechanisms need to be investigated precisely and clearly as a supplementing background of knowledge (1). To address the growing demand for alternative medicine, disease prevention, and disease management, future exploration of novel nutraceuticals and supplements must be continuously created based on the fundamental science of chemistry and biology (2, 3).

The mass production of food employing various new technologies in food processing raised concerns about potential health problems and health issues in the current modernized society and lifestyle. It is not economically feasible to produce specific foods for individuals or small groups of people with a focus on personalized nutrigenomic foods. A multidisciplinary approach and in-depth understanding of food sciences and processing are required for the time- and labor-intensive process of developing personalized food products. It is recommended to consume foods with high nutritional compositions that can be improved based on a person's biological traits and specific goals to develop a personalized approach to food value chains and customized nutrition that meets individual needs. Again, food antioxidants in modulating gut microbial communities are also an interesting topic for developing personalized foods (4). Personalization of food can be emphasized throughout the value chain, from the starting point of food raw materials to the point where the foods are consumed and digested, including understanding the mechanisms of bio-gastronomy and microbiota behavior and interactions. Additionally, it is crucial to investigate the interactions between nutritional content and structural compounds and food processing, as well as combinations of food additives to meet personal requirements (5). Therefore, this Research Topic presents comprehensive and interesting reviews and original research articles related to several aspects relevant to polysaccharides as therapeutic and health-promoting functions in personalized foods including the potential of metagenome-based screening of polysaccharides, the effectiveness of polysaccharides in treating Non-Alcoholic Fat Liver Disease (NAFLD), mushroom β-glucans as functional ingredient, the polysaccharide isolated from *Wolfiporia cocos* as a potential dietary supplement for irritable bowel syndrome (IBS), and also the prevention and treatment of cervical cancer of seabuckthorn polysaccharides.

Bacterial polysaccharides are a significant source of prebiotics and therapeutics. This review discusses the mechanisms of bacterial polysaccharides that exhibit anti-inflammatory, antioxidant, anti-cancer, and anti-microbial properties. Additionally, this report investigates the potential of metagenome-based screening of polysaccharides, the synthetic microbiome-based intervention of polysaccharides as prebiotics, and the commercial applications of microbial polysaccharides (Khan et al.).

Non-alcoholic fatty liver disease is a serious and common condition that can lead to cirrhosis and hepatocellular carcinoma. Currently, there is no approved therapy for this disease. However, preclinical studies have shown that polysaccharides have potential as anti-NAFLD drugs. These studies have demonstrated that polysaccharides can improve glucose and lipid metabolisms, provide antioxidant and anti-inflammatory effects, and regulate the gut-liver axis. The effectiveness of polysaccharides in treating NAFLD is dependent on the chemical structure and characteristics of the polysaccharides, and further research is needed to better understand the structure-activity relationship of anti-NAFLD polysaccharides (Hu et al.).

Mushrooms are considered a superfood with both health-promoting and therapeutic properties. β-glucans, the main contents of polysaccharides, are a dietary fiber with the abilities of anti-diabetic, anti-cancer, cholesterol- and glycogen-lowering properties. The study found that using β-glucan polysaccharide and dried mushroom powder in chicken patties improved moisture retention, maintained cooking yield, and preserved the overall structure of the food matrix. Furthermore, the price and crude fat content of the fat-substituted chicken patties with dried mushroom powder were lower than those with the mushroom-glucan extract. To create low-fat functional foods, it may be advantageous and healthy to use mushroom powder as a fat replacer in food products (see Toh et al.).

Characteristics and properties of a polysaccharide isolated from *Wolfiporia cocos* as a potential dietary supplement for IBS patients were investigated. IBS patients require special care when consuming the FODMAP (Fermentable oligosaccharides, disaccharides, monosaccharides, and polyols) diet, and insufficient dietary fiber intake can have adverse effects on intestinal health. Finding optimum dietary fibers and creating suitable supplements are crucial for IBS patients. To meet the demands for dietary fiber suitable for a low FODMAP diet therapy in IBS, the study discovered that the extract from *Wolfiporia cocos* provides the necessary characteristics and properties, including stability, digestion, viscosity, osmotic activity, adsorption, and fermentation. The study also explored the related mechanisms of the extract, demonstrating that a high *Prevotella* concentration may positively correlate with a high risk of IBS. By supplementing with the extract, there may be an opportunity to prevent intestinal diseases and enhance gut health. Regarding its advantageous properties and characteristics for a low FODMAP diet therapy, the polysaccharide isolated from *Wolfiporia cocos* shows promise as a potential dietary supplement for IBS patients. It may be essential to execute further research and clinical trials to confirm its efficacy and safety (Yang et al.).

Prediction of an active marker of seabuckthorn polysaccharides for the prevention and treatment of cervical cancer, as well as to investigate the mechanisms of action of these polysaccharides has been determined. Seabuckthorn is known to contain various active ingredients, including polysaccharides, flavonoids, tannins, terpenoids, and vitamins, which have been shown to possess many pharmacological activities, such as anti-tumor effects, prevention of cardiovascular and cerebrovascular diseases, anti-inflammation, anti-oxidation, liver protection, and bacteriostasis. In this study, the mechanisms of action of seabuckthorn polysaccharides in the prevention of cervical cancer were investigated using both *in vitro* and *in vivo* experiments. The researchers utilized networks of pharmacological bioinformatics and high-throughput omics analysis to identify the multi-target and multi-pathway action characteristics of these polysaccharides. Overall, the study suggests that seabuckthorn polysaccharides may serve as a promising preventative and therapeutic agent for cervical cancer. Further research is needed to fully understand the mechanisms of action and potential clinical applications of these compounds (Feng et al.).

On behalf of the Guest Editor team, it is our great pleasure to express sincere appreciation for all the contributions to this Research Topic for promoting the global frontier research collaboration.

## Author contributions

YP, WR, PS, and FB wrote the introduction, central part, conclusion, and references. All authors contributed to the article and approved the submitted version.
